# How Do Cultural Factors Influence the Teaching and Practice of Mindfulness and Compassion in Latin Countries?

**DOI:** 10.3389/fpsyg.2017.01161

**Published:** 2017-07-11

**Authors:** Javier García-Campayo, Marcelo Demarzo, Edo Shonin, William Van Gordon

**Affiliations:** ^1^Department of Psychiatry, Aragon Institute of Health Sciences, Miguel Servet Hospital Zaragoza, Spain; ^2^Department of Preventive Medicine, Universida de Federal de Sao Paulo Sao Paulo, Brazil; ^3^Preventive Medicine, Hospital Israelita Albert Einstein Sao Paulo, Brazil; ^4^Awake to Wisdom Centre for Meditation and Mindfulness Research Ragusa, Italy; ^5^Centre for Psychological Research, University of Derby Derby, United Kingdom

**Keywords:** mindfulness, compassion, health, culture, Latin

## Introduction

The first structured 8-week program on mindfulness, Mindfulness-based Stress Reduction (MBSR), was developed by Kabat-Zinn (1982) in a hospital linked to the University of Massachusetts. As is usual in private health systems, participants of these programs have to pay for them, making them less accessible to low-income individuals. Consequently, a large proportion of participants of mindfulness-based interventions have been high-income, white, Anglo-Saxon, and educated individuals actively seeking mindfulness training (Olano et al., 2015). Despite Kabat-Zinn's purported interest in offering mindfulness to low-income populations, few studies have investigated the efficacy and/or acceptability of these programs for individuals of low socioeconomic status (Roth and Creaser, 1997; Kabat-Zinn et al., 2016).

Mindfulness programs are now taught in more than 50 countries worldwide (Kabat-Zinn et al., 2016), including Spanish- and Portuguese-speaking countries of Europe and the Americas (encompassed by the term “*Latin*” in this paper). Such Latin countries share obvious cultural influences and similarities—and during the implementation of mindfulness in these countries—Latin clinicians and researchers have observed that compared to non-Latin countries, there exist differences in how their patients learn and practice mindfulness (Demarzo et al., 2015). In this opinion paper, we briefly provide a preliminary conceptual framework for a culturally-syntonic approach to implementing mindfulness- and compassion-based (M and C) interventions in Latin societies. Furthermore, based on the authors' own clinical and teaching experiences, we offer recommendations for the effective teaching of M and C approaches in Latin countries.

## Cultural factors relating to health system structure

A systematic review of the scientific literature—conducted for the purposes of preparing the present paper (search conducted up until October 2016; *Medline* and *Embase* electronic databases)—demonstrated that no studies on the cultural aspects of teaching and delivering M and C in Latin countries have been conducted to date.

We conducted multiple searches on MEDLINE and EMBASE for references dated between 2015/01/01 and 2017/12/31, using a combination of the following terms: “culture,” “transcultural,” “Latin America,” “Asia,” “Africa,” “poverty,” “low income,” “indigenous,” “deprived,” “population,” “minority groups,” “ethnic groups,” “vulnerability,” “equity,” “meditation,” “mindfulness-based,” “Clinical Trial,” “Guideline,” “Meta-Analysis,” “systematic.” We retrieved 22 references from MEDLINE and 27 from EMBASE.

Despite this research gap, culture, defined as “the complex whole which includes knowledge, beliefs, arts, morals, law, customs, and any other capabilities acquired by a human as a member of society” (Tylor, 1889), clearly influences how individuals relate to both health problems and the treatments formulated to address them. Perhaps the most important cultural difference between US and Latin countries is the structure of, and attitudes toward, the health system. In particular, developed Latin countries (e.g., Spain, Brazil, Chile, Portugal, etc.) possess public health systems which contrasts with the private health system that operates in the US (Demarzo et al., 2015). An overview of how factors relating to health system structure influence M and C programs in US versus Latin countries is shown in Table 1.

**Table 1:** Differences in how health system structure influence M and C programs in US and Latin countries.

**US**	**Latin countries**
Self-funded patients typically from high-income and well-educated backgrounds	Publicly-funded patients typically from middle or low income and/or education levels
Patients typically pay $300–400 per M and C course	M and C training typically provided free of charge (although self-funded is also an option)
M and C training is often proactively sought	Patients typically offered M and C training by their family physician in a passive way

A further key consideration relating to M and C treatment usage is that in Latin countries, adherence-to-practice and compliance levels tend to be lower. A possible explanation is that participants in Latin countries typically do not pro-actively seek M and C training and, due to being publicly funded, do not make any direct financial contribution (and may therefore expect less from the training) (Demarzo et al., 2015). Furthermore, compared to M and C participants in developed countries, participants from Latin countries typically work long hours allowing less time for self-practice or for attending structured mindfulness training sessions. Moreover, in the poorest Latin America countries (such as Bolivia, Central America, etc.), low-income populations often lack basic services, such as water supply and sanitation infrastructure. Consequently, individuals from such populations may not regard training in M and C as a priority, and this may have knock-on effects on adherence and compliance.

## Other health-relevant cultural inclinations in latin countries

Latin countries encompass more than 20 countries on two continents. However, although there are important cultural differences between them, there appear to be common perspectives with regard to their outlook on disease (see García-Campayo and Alda, 2005 and Qureshi et al., 2013 for a more detailed account of the health-relevant cultural inclinations referred to below):

- *Importance of family:* Latin families are usually large, communicate frequently among themselves, and regularly celebrate as a family unit. The family offers an important means of coping with health-related adversity. Informal mindfulness practice in the family environment is frequently reported by individuals from Latin countries (Garcia-Campayo and Demarzo, 2014). However, the need to conform to social norms can increase the risk of manipulation or feelings of responsibility.
- *Expressing feelings*: Latin individuals are more likely to openly reveal and discuss their feelings with friends and relatives. However, this can foster dependence on others' opinions and prompt behaviors that conform to social expectations (García-Campayo and Alda, 2005).
- *Role of body*: Latin individuals typically have fewer reservations in being tactile, showing their bodies, or in making direct eye-contact (i.e., without feeling uncomfortable). This characteristic is almost certainly linked to willingness to express feelings and the benefit of stronger social networks.
- *Health-related guilt*: Health-related guilt appears to be more common in Latin countries (e.g., feeling guilty about being depressed or being diagnosed with an illness).
- *Dependence on primary care physicians and low levels of patient empowerment*: Latin patients can be less eager to know the intricate details of their illness, or to engage with decisions on diagnosis and treatment.

Although the above outlined summary indicates the key health-relevant factors that should be considered when administering M and C approaches to Latin participants, it should be noted that some of these cultural traits can be so deeply embedded in daily life that they are difficult to detect not only by the patient, but also by the clinician. Consequently, prior to implementing the below outlined recommendations, clinicians will obviously need to formulate a detailed clinical picture in order to discern maladaptive and/or pre-disposing attitudes and behaviors from personality traits.

## Recommendations for teaching mindfulness and compassion to latin patients

In light of the above health-relevant cultural factors, the following recommendations – based on the academic literature (e.g., García-Campayo and Alda, 2005; Garcia-Campayo and Demarzo, 2014; Demarzo et al., 2015), consultation with Latin clinicians and M and C participants, as well as the authors' own teaching experiences—are offered to assist both clinicians and researchers using M and C approaches in Latin populations:

1. *Reduce duration of formal practice:* Latin individuals seem to be less committed to M and C formal practice compared to Americans. Consequently, the 45-min home practice recommended in MBSR (Kabat-Zinn, 1982) can be unrealistic. In fact, based on the authors' experience, most Latin participants meditate at home for an average of 20 min per day. Furthermore, audio- or video-guided practices seem to be employed more frequently and for longer durations in Latin versus US populations. Such methods are probably employed to account for the more passive role and lower levels of engagement demonstrated by Latin M and C participants (Olano et al., 2015).
2. *Reduce retreat component:* Most structured M and C programs include a 1-day retreat as standard. However, given that in Latin countries many M and C courses are offered on the public health system, these periods of time can be difficult to justify for service providers. Likewise, from the patient's perspective, a full-day retreat requires a significantly greater time commitment (i.e., potentially conflicting with long working hours and social/family commitments). Reducing or eliminating the full-day retreat component may therefore be beneficial.
3. *Encourage mindfulness of the body:* We have observed that Latin patients (i) often relate easily to body scan or mindful body movement practices and (ii) are typically comfortable in adopting the various meditation postures required for M and C training. Therefore, body-based M and C practices could be emphasized in Latin populations.
4. *Emphasize interpersonal mindfulness:* Given that family plays an important role in Latin society, interpersonal mindfulness (i.e., that necessitates awareness of one's own as well as others' emotions) tends to be well-received by Latin participants. This greater focus on informal/interpersonal practice could explain why, despite less time spent practicing formal meditation, the efficacy of M and C training with Latin participants appears to be comparable with that observed in US populations (Kabat-Zinn et al., 2016).
5. *Be aware of religious cultural influences:* The influence of Christianity in Latin countries warrants consideration. For example, the Spanish and Portuguese word for “compassion” can imply feelings of superiority. Indeed, books on compassion, when translated from English into Spanish or Portuguese, typically avoid this word due to the connotations it engenders. An additional aspect linked to religion is that the Catholic Church may not advocate certain yoga or mind-body practices due to their Buddhist and/or Hinduist influences. Furthermore, the notion of non-duality—that features in certain M and C meditation practices—may not be compatible with Christian beliefs.

## Conclusions and future research directions

M and C approaches are currently being implemented in Latin countries and empirical findings attest to their effectiveness. However, the use of M and C treatments in Latin countries gives rise to a number of cultural challenges. Although the abovementioned recommendations for addressing these challenges are informed by both practice and research, there is a need for targeted empirical investigation in order to better understand (i) the specific cultural differences that influence the effectiveness of M and C approaches in Latin countries, and (ii) how to modify and tailor M and C approaches in order to account for these differences. In a similar manner, there is a need to develop a bank of assessment instruments, validated in the languages and culture of Latin countries, that can be used for research and clinical purposes. Based on our narrative review, the three most different issues affecting the teaching of mindfulness and compassion in Latin countries, in comparison with the UK and US, are the amount of daily practice (this should probably be shorter for Latins), the role of informal practice and interpersonal mindfulness (more important in Latin environments), and the issue of potential religious influences.

## Author contributions

All authors listed have made a substantial, direct and intellectual contribution to the work, and approved it for publication.

### Conflict of interest statement

The authors declare that the research was conducted in the absence of any commercial or financial relationships that could be construed as a potential conflict of interest.
